# Development of forensic mental health services in Japan: working towards the reintegration of offenders with mental disorders

**DOI:** 10.1186/1752-4458-8-21

**Published:** 2014-06-03

**Authors:** Chiyo Fujii, Yusuke Fukuda, Kumiko Ando, Akiko Kikuchi, Takayuki Okada

**Affiliations:** 1 Department of Forensic Psychiatry, National Institute of Mental Health, National Center of Neurology and Psychiatry, 4-1-1 Ogawa-Higashi, Kodaira, Tokyo 1878553, Japan; 2 National Institute of Mental Health, National Centre of Neurology and Psychiatry, 4-1-1 Ogawa-Higashi, Kodaira, Tokyo 1878553, Japan

**Keywords:** Forensic psychiatry, Mentally disordered offenders, Rehabilitation, Social reintegration, Treatability

## Abstract

**Background:**

Until the recent enactment of the Medical Treatment and Supervision Act (MTSA) in 2005, neither legislations nor facilities for mentally disordered offenders were available in Japan. The aim of the country’s forensic mental health services, based on this new law, is to improve the social reintegration of mentally disordered offenders. In order to provide optimal psychiatric care to these individuals, specialised court proceedings, treatment facilities, and concrete guidelines have been established. The aim of this study was to review the current status of the new system and to clarify future challenges for improving services.

**Methods:**

The authors collected official statistics regarding the new system published separately by the Ministry of Health, Labour and Welfare, the Ministry of Justice, and the Supreme Court of Japan. We aggregated the data and examined the system’s current implementation status, nationwide.

**Results:**

There were 2,750 requests for enrolment in the MTSA system between its initiation in 2005 and 31 December 2012. Of those requests, 2,724 cases had been concluded in court. In 63.1% of the cases, an inpatient treatment order had been made; 82.4% of those inpatients were diagnosed with schizophrenia. By the end of March 2012, two patients completing treatment under the MTSA had re-committed a serious offense. While overall designated inpatient and outpatient treatment facilities have reached national targets in terms of resources and beds available, a regional gap in MTSA designated facilities remains and the number of patients under inpatient treatment order is on the increase.

**Conclusions:**

Overall, the MTSA system has been running smoothly without encountering any serious problems. However, several concerns have emerged, such as the accumulation of patients under inpatient treatment order and insufficient regional resources. To more successfully promote the reintegration of mentally disordered offenders, improvements in outpatient treatment and welfare services are crucial. In order to install effective measures to help improve the system, a nationwide database of patients being treated under order of the MTSA should be properly built and maintained.

## Background

Offenders with mental disorders are faced with two significant social disadvantages that make reintegrating into society extremely difficult: the stigma attached to mental disorders and the fact that they have committed an offense. While the spread of the concept of normalisation during the latter half of the 20th century has helped shed light on the importance of rehabilitation and community care for the mentally disordered in many developed countries, mental health services for criminal offenders with mental disorders have not kept pace with these advances.

During this period, Japan was also influenced by the movements of normalisation and deinstitutionalisation. For example, the Mental Health Law was revised in 1987, emphasising the protection of human rights and rehabilitation of patients with mental disorders. This was followed by the Mental Health and Welfare Act (subsequently, MHWA) of 1995, which emphasised patients’ welfare and promotion of their self-reliance [1].

However, with regard to forensic mental health services, neither legislations nor facilities for mentally disordered offenders were available in Japan until recently. When mentally disordered offenders were found to be insane, or given a reduced punishment without imprisonment on account of diminished mental capacity, they were typically treated as involuntarily admitted patients in ordinary psychiatric hospitals alongside non-offender patients.

There are two forms of involuntary civil admission in Japan [1]: ‘admission for medical care and protection’ and ‘administrative involuntary admission’. The former applies when a patient does not consent to admission, despite an MHWA-designated psychiatrist having ruled that they suffer from a mental disorder requiring inpatient treatment. In these instances, ‘the person liable for protection of the patient’ , who is designated by a domestic court, can give consent for admission. On the other hand, the latter involves the involuntary admission of an individual by order of the prefectural governor. This applies when two MHWA-designated psychiatrists independently conclude that the patient poses an imminent risk of harm to himself or herself, or others, due to a mental disorder. Until 2005, when a new law regarding how to deal with serious offenders with mental disorders came into being, these forms of involuntary civil admission would have applied to persons with mental disorders who had committed a serious offense.

In Japan, involuntary civil admission to a psychiatric hospital is not adjudicated by tribunals. Therefore, historically, psychiatrists in charge of offender patients were responsible for making decisions concerning not only their treatment but also when they could be discharged from hospital, even though they had committed a serious offense. Additionally, there was no management system in place to ensure a patient’s compliance with psychiatric treatment after discharge. Moreover, more than 80% of Japan’s psychiatric facilities consist of private hospitals [2] and, as such, are insufficiently equipped, security-wise, to treat criminal offenders.

In order to improve this problematic situation, the Act for the Medical Treatment and Supervision of Persons with Mental Disorders Who Caused Serious Harm (subsequently, the Medical Treatment and Supervision Act; MTSA) was finally passed in 2003, after decades of debate. The new law laid out conditions as well as procedures for the management of serious offenders with mental disorders, and aimed to promote the rehabilitation of persons who had caused a serious offense in a state of insanity or diminished capacity, thus reflecting current trends of normalisation and deinstitutionalisation of the mentally disordered. In accordance with this aim, the law established specific rules on the management of serious offenders with mental disorders, and provided such persons with appropriate, continuous medical treatment and supervision under the responsibility of the government. This law came into effect on 15 July 2005; it was genuinely the beginning of the forensic mental health services era in Japan.

### Implementation of the MTSA system

**Figure 1:**
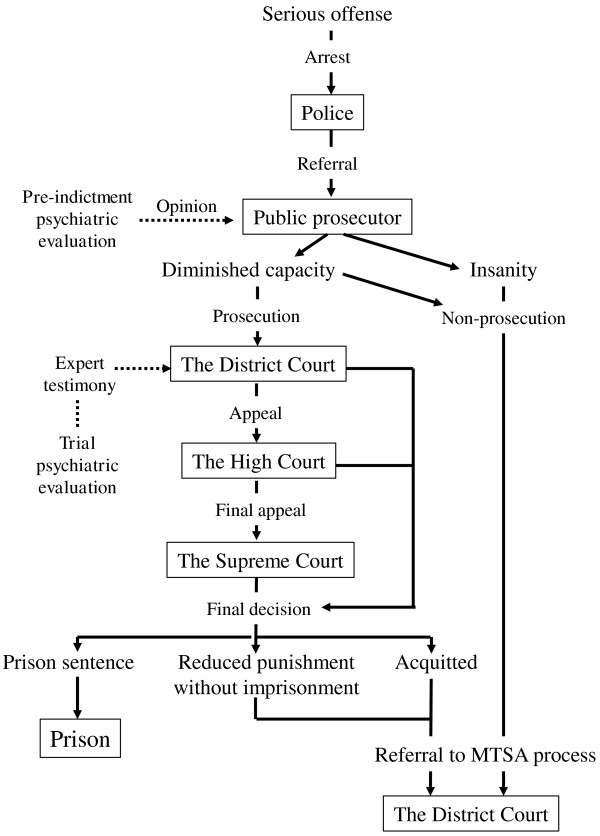
Flow of the referral process to the Medical Treatment and Supervision Act (MTSA) system.

The MTSA applies specifically to mentally disordered individuals who have committed a serious offense, such as homicide, arson, robbery, rape, forcible indecency (including attempts at these offenses), and injury. There are two primary situations in which the MTSA is implemented. First, public prosecutors can request that the MTSA be applied if they decide not to charge someone who has committed a serious offense on the grounds of insanity or diminished mental capacity, typically based on the results of the pre-indictment psychiatric evaluation. Alternatively, a person who has been acquitted or given a reduced sentence without imprisonment in an ordinary criminal trial by reason of criminal irresponsibility or diminished responsibility can be referred to the MTSA process. Criminal responsibility is determined by three professional judges and six citizen-judges who give probative weight to the results of the trial’s psychiatric evaluation. Figure 1 illustrates the processes involved in requesting the District Court to include an offender in the MTSA system. All individuals are supposed to be accompanied by a lawyer during referrals to Court to ensure that their rights are protected.

### MTSA psychiatric evaluation

Following referral by the public prosecutor, the District Court orders a psychiatric evaluation of the referred individual as part of the court process (the MTSA psychiatric evaluation), which is performed in a specialised inpatient setting and must be conducted within three months. The primary purpose of the MTSA psychiatric evaluation is to verify the following:

1. Presence of mental disorder: Is the mental disorder that caused insanity or diminished capacity at the time of the offense still present?

2. Treatability: Is the individual with the mental disorder expected to respond to medical treatment?

3. Factors impeding the person’s reintegration into society: Are there any factors that would impede the said person’s rehabilitation without ensuring against the recurrence of similar acts?

Whether an inpatient or outpatient treatment order is made in court depends on the extent to which an individual meets all three of these criteria. The referred individual is observed and evaluated by a multi-disciplinary team at the hospital while receiving any necessary medical treatment. A report on the results of this evaluation is then submitted to the District Court to aid in the decision as to whether treatment under the MTSA is necessary for the referred individual.

### Decision making in the district court

An interdisciplinary panel consisting of a judge and a specially qualified psychiatrist is set up in the District Court when the MTSA psychiatric evaluation is ordered. Additionally, a rehabilitation coordinator who is on the staff of the probation office is assigned to each referred individual. Rehabilitation coordinators are qualified mental health professionals (typically, psychiatric social workers) with substantial experience in the fields of mental health and welfare who investigate the individual’s social circumstances and submit the results to the District Court.

Based on the MTSA psychiatric evaluation results and the rehabilitation coordinator’s report on the individual’s social circumstances, as well as the testimony of the individual, his/her lawyer, and the public prosecutor, the members of the District Court panel determine the optimal way to handle the individual. Opinions from the psychiatrist’s medical perspective carry the same weight as those from the legal perspective, and the panel can request a qualified mental health advisor to evaluate the individual’s condition from a mental health and welfare point of view.

**Figure 2:**
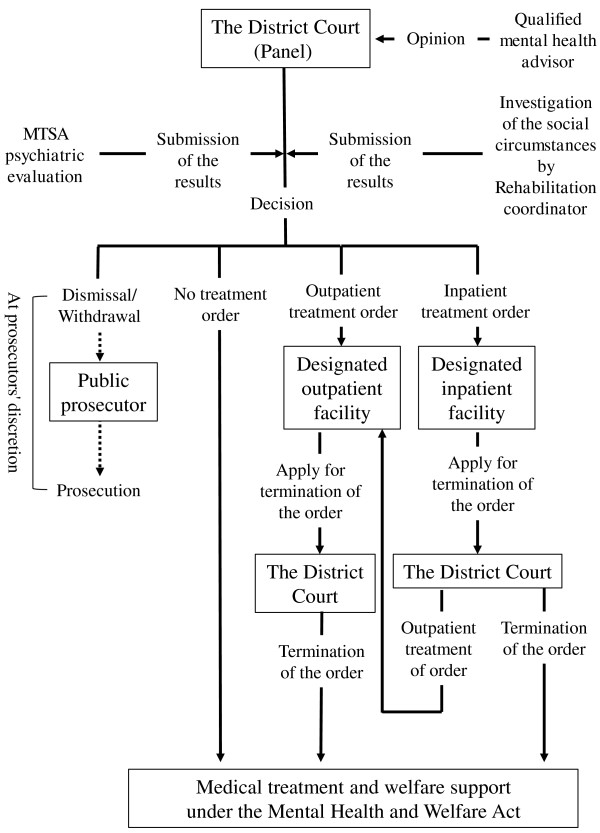
The decision-making process from referral to termination under the Medical Treatment and Supervision Act (MTSA).

If the panel finds that treatment under the MTSA is necessary to improve the referred individual’s mental condition and aid in social reintegration without reoffending, the District Court can order the individual to undergo either inpatient or outpatient treatment. In cases where the panel decides that the referred individual does not meet criteria for treatment under the MTSA, the individual is given the ruling of ‘no treatment order’. However, this does not imply that psychiatric treatment is unnecessary; rather, these individuals may require psychiatric treatment under the MHWA instead of the MTSA and are thus treated as involuntary patients alongside non-offender patients.If the refereed individual is found to be neither insane nor having diminished mental capacity, the case may be dismissed from the District Court. Alternatively, the same judgment may be reached if it is ruled that the individual did not commit a serious offense. Finally, the public prosecutor may withdraw the referred case before a judgment for any reason. In cases that are dismissed or withdrawn, the referred individual can be charged at the discretion of the public prosecutor. Figure 2 delineates the progression from referral to termination under the MTSA.

### Inpatient treatment order

Individuals who are given an inpatient treatment order are to be admitted to a forensic unit in an MTSA designated secure facility managed by the state, local municipalities, or public corporations. The facilities are equipped with standardised security management systems, as well as sufficient human resources. Multi-disciplinary teams provide a tailored treatment programme for each patient in accordance with the Guidelines for the Inpatient Treatment of the Medical Treatment and Supervision Act [3].

Inpatient facilities must have a system in place by which they can be appraised by outsiders, and they must hold liaison conferences at regular intervals with mental health professionals working in the community. An evaluation of the treatment provided is also conducted regularly, and when a patient recovers well enough to be discharged, the director of the facility must promptly apply to the District Court for the termination of the individual’s inpatient order. If a patient requires prolonged hospitalisation, the director of the facility must request a continuation of the inpatient order from the court every six months.

### Outpatient treatment order

There are three types of services afforded to individuals under an outpatient treatment order: (1) mental health supervision by the probation office; (2) medical treatment provided by MTSA designated psychiatric facilities, including hospitals and ambulatory clinics; and (3) social welfare services provided by a mental health centre and support facilities for the mentally disabled in the community where the patient lives.

Rehabilitation coordinators play a key role throughout the duration of the outpatient treatment order. They develop an implementation plan for the patient, and they facilitate effective collaboration and coordination among the relevant authorities and institutions to ensure continuous medical treatment for the patient under their charge. They also work to create a better community environment for the patient to promote their rehabilitation.

MTSA designated psychiatric hospitals and clinics must hold a multi-disciplinary meeting once a month within each facility to assess the patient’s condition and review the goals and policies of his/her treatment. A care programme approach (CPA) conference should also take place at regular intervals, in which all concerned parties, including the patient, his/her family, the multi-disciplinary team, mental health professionals in the community, and the rehabilitation coordinator, participate.

Under an outpatient treatment order, the patient has a legal obligation to live in a stable location and to appear at the probation office at the office’s request. The duration of the outpatient treatment order is typically less than three years; however, if the patient is determined to still require mental health supervision after this time, the District Court can add up to two additional years to their outpatient treatment order. If the patient’s condition has escalated to the level of requiring re-admission to a MTSA designated secure facility, the District Court can give him/her an inpatient treatment order. If this is a temporary aggravation in condition, the patient may be admitted to an ordinary psychiatric hospital as a voluntary or involuntary patient under the MHWA instead of being given an inpatient treatment order.

### Continuing support after termination of the treatment order

When the District Court finds that the medical treatment and supervision of the patient under the MTSA is no longer necessary, or when the specified period for the outpatient treatment order expires (usually after three years; up to a maximum of five years, if necessary), a decision must be made as to whether the patient should continue receiving medical and welfare support. In most cases, continued support is considered to be of crucial importance to empower the patient to lead a life of his/her own in the community. Thus, in order to achieve an unimpeded transition from receiving medical treatment and supervision under the MTSA to medical and welfare support under the MHWA, it is necessary to adopt a realistic approach, taking into account the situations surrounding the patient after termination of the order.

### Objection from a patient

A patient under the MTSA is allowed to file an appeal against the District Court’s order to the high court within two weeks of the judgment; after the allotted period for filing an appeal, he/she retains the right to request a termination of the order from the District Court. The patient is informed of this right and the procedures for objection in a way that is accessible and comprehensible in available booklets. The patient’s family, his/her lawyer, the probation office, and the administrator at the designated inpatient facility may also raise an objection against an order or treatment.

The aim of the present study was to ascertain the MTSA system’s current implementation status and clarify future challengers for improving its services by reviewing available official data available.

## Methods

Data related to the MTSA system were obtained from official statistics released by relevant government ministries.

Court decisions by type of offense were collated from facts and figures presented in the White Paper on Crime (15 Jul. 2005–31 Dec. 2012) [4]. Data on clinical diagnoses of patients under inpatient treatment orders were extracted from the latest official statistics of the Ministry of Health, Labour and Welfare (31 Dec. 2013) [5]. Annual variations in the numbers of patients treated under MTSA order were obtained from the Annual Report of Statistics on Rehabilitation (15 Jul. 2005–31 Dec. 2012) [6] and Judicial Statistics (15 Jul. 2005–31 Dec. 2012) [7]. The recidivism rate for serious offenses among persons who had completed treatment and supervision under the MTSA was derived from the Annual Report of Statistics on Rehabilitation (15 Jul. 2005–31 Dec. 2012) [6] and the report published by the Ministry of Justice and Ministry of Health, Labour and Welfare in July 2012 on the MTSA’s implementation status [8]. Regional resource allocation gaps were calculated from the number of MTSA designated facilities published by the Ministry of Health, Labour and Welfare on 31 December 2013 [9] and data gleaned from the Population Survey Report (31 March 2012) [10]. Medical care expenditures accrued for inpatient and outpatient treatment under the MTSA were estimated from the budget framework for the 2014 fiscal year [11].

Under the Ethical Guideline of Epidemiological Research established by the Ministry of Health, Labour and Welfare [12], the current study, using only available official statistics with anonymous samples, was classified as research requiring no consultation with the ethical review board.

## Results

### Details of the court decisions

Between 15 July 2005 and 31 December 2012, there were 2,750 requests for enrolment in the MTSA system. Of those requests, 2,724 cases had been concluded in court.

Table 1 details court decisions based on type of offense, from the enactment of the MTSA in 2005 to the end of 2012. Inpatient orders were prescribed most often, accounting for 63.1% of the decisions. When examining court decisions by type of offense, orders for inpatient treatment were also highest for each individual offense. No treatment was ordered in 17.0% of all court decisions, which was larger than the percentage of outpatient treatment orders.

**Table 1:** Court decisions by types of offense (15 July 2005–31 Dec. 2012)

	**Court decision**					
	**Inpatient order N (%)**	**Outpatient order N (%)**	**No treatment order N (%)**	**Dismissal N (%)**	**Withdrawal N (%)**	**Total N**
Arson	430 (57.3%)	151 (20.1%)	152 (20.3%)	17 (2.3%)	0 (0%)	750
Rape/forcible indecency	82 (57.3%)	18 (12.6%)	33 (23.1%)	7 (4.9%)	3 (2.1%)	143
Homicide	536 (70.6%)	98 (12.9%)	108 (14.2%)	11 (1.4%)	6 (0.8%)	759
Injury	593 (64.1%)	133 (14.4%)	143 (15.5%)	49 (5.3%)	7 (0.8%)	925
Robbery	78 (53.1%)	25 (17.0%)	28 (19.0%)	13 (8.8%)	3 (2.0%)	147
Total	1719 (63.1%)	425 (15.6%)	464 (17.0%)	97 (3.6%)	19 (0.7%)	2724

### Clinical features of inpatients

Table 2 describes the clinical diagnoses of those receiving inpatient treatments through 31 December 2013. Males accounted for 75.4% of 743 total inpatient orders, and the most common diagnosis was schizophrenia, comprising 82.4% of all inpatient cases.

**Table 2:** Clinical diagnoses of individuals under inpatient treatment order

		**Male**		**Female**		**Total**	
		**N (%)**		**N (%)**		**N (%)**	
F0	Organic disorders	10	(1.8)	1	(0.6)	11	(1.5)
F1	Substance use	44	(7.9)	6	(3.3)	50	(6.7)
F2	Schizophrenia	467	(83.4)	145	(79.2)	612	(82.4)
F3	Mood disorders	15	(2.7)	22	(12.0)	37	(5.0)
F4	Neurotic disorders	1	(0.2)	5	(2.7)	6	(0.8)
F6	Personality disorders	6	(1.1)	2	(1.1)	8	(1.1)
F7	Mental retardation	6	(1.1)	2	(1.1)	8	(1.1)
F8	Disorders of psychological development	10	(1.8)	0	(0.0)	10	(1.4)
G4	Epilepsy	1	(0.2)	0	(0.0)	1	(0.1)
Total		560		183		743	

### Number of patients under MTSA order and designated facilities

Table 3 details the numbers of cases treated under MTSA order that were commenced, terminated, or readmitted. By the end of March 2012, two persons had re-committed a serious offense and were referred to the District Court for re-enrolment in the MTSA system—accounting for 0.3% of the total number of persons who had completed treatment under the MTSA by the end of December 2011.

**Table 3:** Annual variations in numbers of patients under MTSA order

	**Inpatient treatment (N)**				**Outpatient treatment and mental health supervision (N)**			
	**Commencement of inpatient treatment**	**Discharge and commencement of outpatient treatment**	**Termination of MTSA treatment**	**Under inpatient treatment order**	**Commencement of treatment and supervision**	**Termination of MTSA treatment**	**Under outpatient treatment order**	**Readmission**
2005*	47	0	-	47	19	-	19	0
2006	191	28	2	206	119	16	122	1
2007	253	73	22	360	188	30	247	1
2008	259	114	28	474	178	61	364	2
2009	210	166	47	469	223	122	465	5
2010	246	151	32	529	216	157	524	5
2011	280	140	23	642	152	176	530	12
2012	263	188	41	668	235	215	550	6

2005*:15 July 2005–31 December 2005.

As of 31 December 2013, there were 30 designated inpatient facilities in Japan, each with 5 to 66 psychiatric beds available, for a total of 791 beds across all facilities. Table 4 shows the number of beds in the designated inpatient and outpatient facilities by geographic region.

**Table 4:** Number of MTSA designated facilities by region (31 Dec. 2013)

	**Designated outpatient facilities**		**Designated inpatient facilities**	
	**Number of facilities (N)**	**Per 1,000,000 population**	**Number of beds (N)**	**Per 1,000,000 population**
Hokkaido region	38	6.94	0	0
Tohoku region	51	5.52	33	3.57
Kanto region	88	2.1	299	7.14
Chubu region	69	3.21	139	6.47
Kinki region	83	3.7	106	4.72
Chugoku region	34	4.52	91	12.92
Shikoku region	27	6.76	0	0
Kyushu region	62	4.24	123	8.41
Total	452		791	

### Medical care expenditure

The results on medical care expenditure are shown both in Japanese Yen (JPY) and U.S. dollars (USD). Purchasing power parity between JPY and USD in 2014 (USD 1 = JPY 101.7) was used to calculate the USD equivalent of the cost. All medical expenditures accrued for inpatient and outpatient treatment under the MTSA were publicly funded. The Ministry of Health, Labour and Welfare allocated JPY 18,922,726 (USD 186,019) for each patient undergoing inpatient treatment and JPY 1,447,627 (USD 14,230) for patients with outpatient treatment orders in the 2014 fiscal year budget.

## Discussion

### Changes in mental health services after initiation of the MTSA

Up until 2005, mentally disabled persons who had committed a serious offence were treated under the framework of general psychiatry in Japan [13]. This was a unique situation, particularly when compared with European nations; for example, the German Penal Code, revised in 1933, specifies that those who are found incompetent to stand trial or declared not criminally responsible, as well as those who are considered to have diminished responsibility and who may be expected to commit further serious crimes, are placed involuntarily in forensic hospitals. Additionally, the ‘Dangerous Habitual Offenders and their Detention and Rehabilitation Act’ , introduced in the same year, outlines procedures to deal with ‘dangerous recidivists’ , including persons with mental disorders [14]. Although Japan’s Penal Code has been strongly influenced by the German Penal Code, it only includes a simple article on the relationship between mental capacity and criminal responsibility, stating ‘an act of insanity is not punishable; an act of diminished capacity shall lead to the punishment being reduced’. The French criminal code bears greater resemblance to that of Japan, as, until recently, there were no laws there that included social-defence theories for mentally disordered offenders [15]. However, ‘Difficult Patient Units’ have been in place in France since 1910, accepting mentally disordered offenders, as well as aggressive and/or violent patients referred from general psychiatric hospitals.

Mental health services in Japan have centred on hospital-based care since the end of World War II, when involuntary long-term admissions were common even among non-offender patients. At that time, as described in the Background, psychiatrists had full responsibility for making decisions on how to deal with such patients. Thus, psychiatrists dealing with difficult patients who had committed a serious offense often treated them by hospitalizing them for longer than necessary as a safety measure to keep them from reoffending.

The MTSA brought about substantial changes to this situation, and treatment under the MTSA is subject to periodic judicial review by the District Court. This process ensures transparency of the treatment prescribed and should prevent unnecessarily long hospitalisations. Additionally, given the perception that deinstitutionalisation could increase the need for forensic mental health services [16], setting up appropriate measures to treat offenders with mental disorders could be essential in the promotion of the government policy ‘Visions in Reform of Mental Health and Medical Welfare’ [17], emphasising the necessity of transitioning from hospital-based to community-based care.

### Problems concerning ‘treatability’ and ‘risk of recidivism’

From the introduction of the MTSA system through the end of 2012, 2,724 cases were resolved in the District Court as the results shows. It is notable that ‘no treatment orders’ account for a large proportion of the rulings. The reason for this is considered to be that the panels of the District Court place emphasis on ‘treatability’ out of the three guiding criteria for treatment orders in their decisions.

Medical treatment and supervision under the MTSA are highly multidisciplinary, providing patients with intensive care, but at an extremely high cost. As the results on medical expenditures show, the cost for each patient undergoing inpatient treatment is estimated to be JPY 18,922,726 (USD 186,019) a year, which means that the average cost per day amounts to approximately JPY 51,843 (USD 510). This is about 4.4 times higher than the cost of administrative involuntary admission [18].

Considering the expense and limited budget available, there is speculation that it is more rational to put greater resources into patients who are most effectively treated. A lucid ‘untreatable’ example is offenders with advanced dementia, where the most appropriate form of care might be placing them in a nursing home rather than providing them with intensive medical treatment in a high security unit. The same can be said regarding offenders with mental retardation and disorders of psychological development. As data presented in Table 2 reflects, the proportion of patients with these disorders under inpatient treatment order is low. On the other hand, it has been argued that the MTSA system should be applied to patients who are less treatable precisely because they are difficult to help within the framework of conventional mental health services.

In this context, the fundamental question arises as to who would be eligible for treatment within the MTSA system. As is the case with mental retardation, orders for inpatient treatment are rare for individuals with personality disorders, accounting for only 1.1% of the total (see Table 2). This finding contrasts with those on other countries, in which offenders with personality disorders have been noted to comprise the main clientele of forensic mental health services. For example, in Germany, 37% of inpatients at the Haina Forensic Psychiatric Hospital have been reported to have personality disorders [19]. The reason for this discrepancy is clear. In Japan, offenders with personality disorders are mostly found to be able to take full criminal responsibility before they arrive at the stage where they are actually referred to the MTSA system. This means that offenders with personality disorders are seldom referred to the MTSA system to begin with. They are often subject to a prison sentence and receive medical treatment in correctional facilities if necessary.

The structural problems within the system will be discussed further in this section, but first, if the primary purpose of the MTSA were to protect society from ‘dangerous recidivists’ , the system would have to accept offenders with personality disorders by setting up measures that would enable such offenders to be transferred from correctional facilities to the MTSA designated facilities, even if they have been found to be able to take full criminal responsibility. The enactment of the current law on how to deal with offenders with mental disorders was greatly accelerated by an act of atrocity several years ago involving an individual with a personality disorder who had previous criminal records and a history of admissions into psychiatric hospitals, including ‘administrative involuntary admission’ with a diagnosis of schizophrenia. This individual broke into an elementary school in the daytime, in June of 2001, fatally stabbing eight pupils and injuring thirteen others and two teachers. Even though a psychiatric evaluation later concluded that the said individual had a personality disorder instead of schizophrenia [20] and he was subject to capital punishment after being found to be able to take full criminal responsibility at the trial, this incident evoked public reaction calling for legislation regarding offenders with mental disorders and the risk of recidivism. Nevertheless, the present structure of the MTSA system, which was eventually established following this incident, is not as receptive of offenders with personality disorders as one might anticipate. Moreover, there is no clause in the criteria for admission into the MTSA system referring to the assessment of recidivism risk. This situation is somewhat baffling, given the event which triggered the law’s establishment in the first place.

In fact, references were made to ‘recidivism risk’ in the initial stages when the bill was being presented to Parliament. However, the word ‘risk’ was subsequently deleted from the final bill. Lawmakers avoided using the word ‘risk’ in the MTSA due to the law’s historical precedents [21]. Since the 1920s, attempts have been made to establish new laws dealing with offenders with mental disorders. These attempts were hampered in the 1940s by World War II, and continued to face strong opposition in the post war era during a time of heightened patients’ rights, the evolution of antipsychiatry, and the increasing move towards normalization and deinstitutionalization. Opposition groups called for prudence out of concern that a law stipulating how to deal with offenders with mental disorders could lead to proactive detention of individuals with mental disorders, on the grounds of their ‘risk’ of criminal offense, for the safety of the public. In view of this concern, the decision was finally made to refrain from using the word ‘risk’ in the text of the new law, as it might evoke notions of ‘measures for the safety of the public’. While the expression ‘without recurrence of similar acts’ in the third criterion can be interpreted to imply the necessity for such assessment, this ambiguous description undoubtedly causes confusion in clinical practice, where the ‘risk for suicide and offensive behaviour’, which differs delicately from the risk of recidivism, is routinely assessed. More in-depth discussions on this issue, both from a practical and ethical standpoint, should be conducted in the future.

The fact that offenders with personality disorders are seldom referred to the MTSA system is of great relevance to its low recidivism rate. From the standpoint of recidivism prevention, the MTSA system could perhaps consider accepting such offenders in a more positive manner. However, given the MTSA system’s potential for improving psychiatry in general—through the applicability of its sophisticated methods and upgrading of community mental health care systems for persons under outpatient treatment order—priority might be given, for the time being, to improving the current general mental health care system for ‘treatable’ patients (as typified by patients with schizophrenia who have been inappropriately treated in the absence of a proper forensic mental health care system), over developing a system specifically for dealing with offenders with personality disorders.

### Resource allocation

As shown in Table 3, the number of patients treated under order of the MTSA is gradually increasing. As of 31 December 2012, 668 persons were under inpatient treatment order in Japan (at the time, Japan’s population was estimated to be 127,515,000). This figure was relatively small in comparison with those in other developed countries—England and Wales, for example, or Germany. According to official statistics published in 2008 [22], a total of 3,937 offenders with mental disorders were detained in hospital as of 31 December 2008 in England and Wales, the population of which was approximately half that of Japan. In Germany, which has a population roughly two-thirds of Japan’s, 6,287 inmates were reported to be receiving treatment in forensic hospitals in 2008 [13]. Even though it is increasing, the small number of offenders under inpatient treatment order in Japan is believed to be partly attributable to the stringent conditions for commencement of the MTSA system, which is aimed at only those who are referred to the system by the public prosecutor and who meet the three criteria described in the Background. Nevertheless, comparisons between these various systems are difficult because of the vast differences that exist in the countries’ legal structures.

Although there are no official statistics regarding the average duration of inpatient treatment, the previous research estimated it to be 608 days based on data collected from patients admitted to the designated MTSA inpatient facilities between 15 July 2005 and 14 July 2011 [23]. Initially, the government assumed that the average length of an inpatient treatment would be 18 months; however, another study on patients receiving inpatient treatment has indicated that the average duration of inpatient treatment is increasing over time [24]. Given the discrepancy between overly optimistic estimates and previous research findings, and the fact that the number of cases under inpatient treatment orders is gradually increasing, the accumulation of long-term patients is likely to become a major problem. Ongoing research is essential to determine factors contributing to this trend and establish effective measures to address it.

There are currently 791 beds available nationwide in designated MTSA inpatient facilities, surpassing the original target of 720 beds dedicated to forensic mental health services. However, there are still large regional differences in resource allocation, as depicted in Table 4. Although individuals assigned to inpatient treatment may be allowed to be admitted to a hospital located in a region other than where they will live after being discharged, treatment in a hospital far away from their residence is undesirable given the difficulty in coordinating community care after discharge. According to the Guidelines for the Inpatient Treatment of the Medical Treatment and Supervision Act [3], patients under the inpatient treatment order are required to undergo training for a period of time to prepare them for real-life situations before being discharged, with the assistance of staff at the designated MTSA inpatient facilities where they have been admitted. If the regions to which patients will return upon discharge have no designated MTSA inpatient facilities, they will have to receive this training at a large distance from the facilities where they have been treated. This poses a heavy burden on both patients and inpatient facility staff. Further, since all costs pertaining to the training are publicly funded, including staff travel expenses, regional differences in resource allocation will lead to financial strain.

Insufficient community resources available for providing appropriate care to individuals in outpatient treatment are another common problem [21]. As shown in Table 4, the number of cases under outpatient treatment orders is on the increase, and according to previous research, the length of treatments completed under outpatient order averaged 495.4 days [25]. Whereas designated inpatient facilities are under the control of national or local governments, more than 80% of outpatient facilities are private institutions with inadequate human and financial resources [25]. Additionally, outreach services for the mentally disordered are not highly developed in Japan [26], meaning community crisis intervention is difficult to provide effectively. Regarding welfare services, long-stay facilities that provide life skills training, such as group homes, are entitled to receive incentive remuneration for accepting individuals under outpatient treatment orders. In addition, expenses incurred in the provision of community life support services conducted by municipalities are subsidised by the government. However, there is currently no incentive compensation available for other types of welfare services accepting offenders with mental disorders. In order to more successfully promote the reintegration of mentally disordered offenders, improvements in outpatient treatment and welfare services are essential. This could also help to raise the quality of mental health services available to the community as a whole, beyond that of forensic mental health services.

### Structural problems within the system

Under the current legal system, it is not possible to transfer offenders with mental disorders who are presently in correctional facilities into the MTSA system. Although these individuals can seek psychiatric treatment in a medical prison, there is a serious shortage of mental health care resources in correctional facilities [27]. According to the Code of Criminal Procedure, if the psychiatric condition of a prisoner is too critical to continue the execution of a sentence, he/she will be suspended from its execution at the discretion of the public prosecutor and be admitted to an ordinary psychiatric hospital for treatment under the MHWA. In such cases as well, the individual would not be allowed to be admitted to a designated MTSA inpatient facility. Similarly, if the accused offender is in a mental state deemed unfit for trial, proceedings will be suspended after the opinions of the public prosecutor and lawyer have been heard. Following this, the said individual will be sent to an ordinary psychiatric hospital instead of a designated MTSA facility. Therefore, it is vital to develop appropriate measures to deal with mentally disordered offenders who are currently imprisoned and accused offenders who are unfit to stand trial.

Sufficient consideration has been made with regard to the human rights of patients under MTSA order by due process of law, as they are required to be accompanied by a lawyer from the beginning of the process, ensuring that patients and their families have the opportunity to raise an objection against an order or treatment. Additionally, an ethical committee regarding the appropriateness of treatment meets regularly (twice a month) and as needed [3]. The most serious ethical dilemma related to forensic mental health services in Japan may be that of justice in resource allocation. Under the current situation, considerably more personnel and medical resources have been invested into the MTSA system than general psychiatric services and mental health care in correctional facilities. From the standpoint of patients’ rights to medical care, it is important to strike the appropriate balance in resource allocation among the different types of mental health care services regulated by different laws.

While there are still lingering problems in the forensic mental health system in Japan, the MTSA has proven to be effective judging from the low recidivism rate. However, the present data is insufficient to allow a comprehensive evaluation of the new system in terms of the primary purpose of the law, which is to improve offender reintegration. In addition, despite the government’s efforts to inject a significant amount of public funds into the system, few cost-benefit analyses have been conducted. Until now, there have not been any evaluations conducted that have collected data on the entire forensic mental health system and analysed its outcomes. In fact, with the current data available, it is difficult to even determine the number of suicides that have occurred among patients treated under the MTSA. Basic information regarding recidivists is also undisclosed, including information on their diagnoses. Patient confidentiality is clearly of capital importance; however, the appropriate disclosure of information while giving due consideration to the protection of personal information is also important in improving the quality of forensic mental health services. Given the current context, a practical measure with which to gather data from all designated facilities should be appropriately developed and maintained at the initiative of administrative agencies, with the goal of improving forensic mental health services.

Another significant issue that needs to be addressed in efforts to promote the reintegration of offenders with mental disorders into society is the need to strengthen current support structures for victims of crime and their families. Despite recent improvements in these public support systems [28], not enough is being done to meet the needs of victims and their families. The initiation of a mental health care system for offenders, without an accompanying improvement in support systems for victims, could be regarded as unjust.

## Conclusions

Since its introduction in 2005, the newly established forensic mental health care system in Japan, based on the concept of normalisation, has been running smoothly without any serious problems. However, there are still various issues to consider, including a discrepancy in regional resources, the accumulation of patients under inpatient treatment order, and the need for appropriate measures to deal with mentally disordered offenders who are currently imprisoned and accused offenders who are unfit to stand trial. To more successfully promote the reintegration of mentally disordered offenders into society, improvements in outpatient treatment and welfare services are crucial. In order to install effective measures to help improve the system, a nationwide database of patients being treated under order of the MTSA should be properly built and maintained.

## Abbreviations

MTSA: Medical treatment and supervision act; MHWA: Mental health and welfare act; JPY: Japanese yen; USD: U.S. dollar.

## Competing interests

The authors declare that they have no competing interests.

## Authors’ contributions

CF analyzed and interpreted the data, and wrote the first draft of this paper. KA and AK contributed to subsequent draft. UF conceived the study, assisted with drafting the paper. TO assisted with data interpretation and drafting the paper. All authors read and approved the final manuscript.
